# Supplementary figures and images for: Evaluation of a multiplex PCR method for the detection of porcine parvovirus types 1 through 7 using various field samples
Source: PLoS One. 2021 Jan 28;16(1):e0245699. doi: 10.1371/journal.pone.0245699 (PMC7842984; doi:10.1371/journal.pone.0245699)

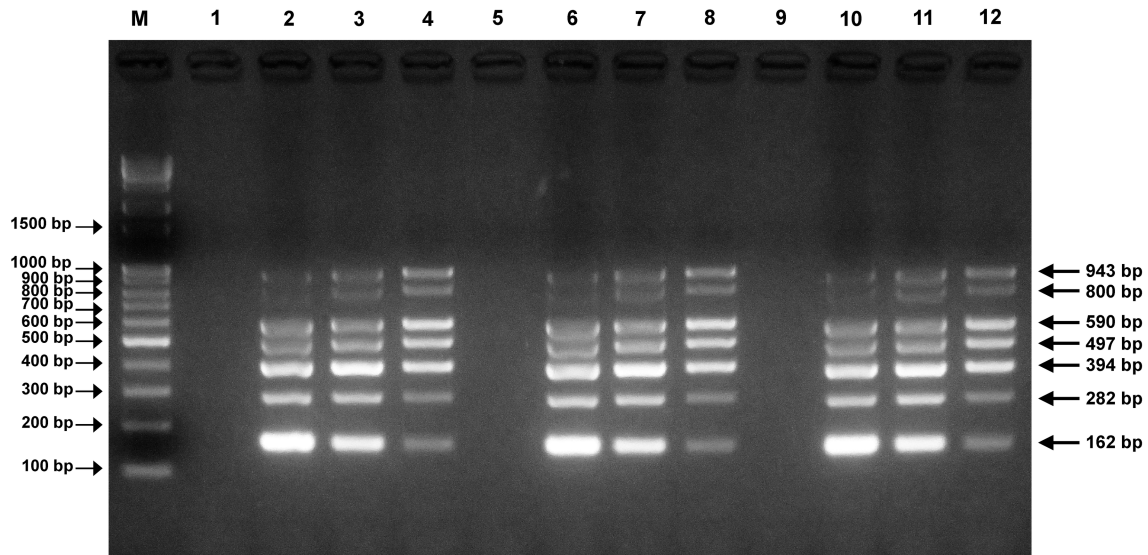

Supplement: S1 Fig — Mixed PPV plasmids were diluted as templates from 3×10^5^ to 3×10^3^ copies/μl to amplify specific fragments by using three different PCR instruments at different times. Lane M, 100-bp plus DNA ladder; lanes 1, 5, and 9, negative controls for the three tests; lanes 2~4, mPCR amplification of 3×10^5^ copies/μl, 3×10^4^ copies/μl, and 3×10^3^ copies/μl PPV plasmids; lanes 6~8 and 10~12, repeated mPCR. (PDF) [file pone.0245699.s001.pdf]
