# Supplementary material for: Evaluation of a multiplex PCR method for the detection of porcine parvovirus types 1 through 7 using various field samples
Source: PLoS One. 2021 Jan 28;16(1):e0245699. doi: 10.1371/journal.pone.0245699 (PMC7842984; doi:10.1371/journal.pone.0245699)
Supplement: S2 Fig — Eighty serum, 40 lung/lymph node and 40 intestine/fecal samples were tested with the established mPCR method. (A) Serum samples: lane M, 100 bp-plus DNA ladder; lane PC, positive controls; lane NC, negative controls; lanes 1~80, mPCR-tested serum samples. (B) Lung/lymph node samples: lane M, 100 bp-plus DNA ladder; lane PC, positive controls; lane NC, negative controls; lanes 1~40, mPCR-tested lung/lymph node samples. (C) Intestine/fecal samples: lane M, 100 bp-plus DNA ladder; lane PC, positive controls; lane NC, negative controls; lanes 1~40, mPCR-tested intestine/fecal samples. (PDF) [file pone.0245699.s002.pdf]

A

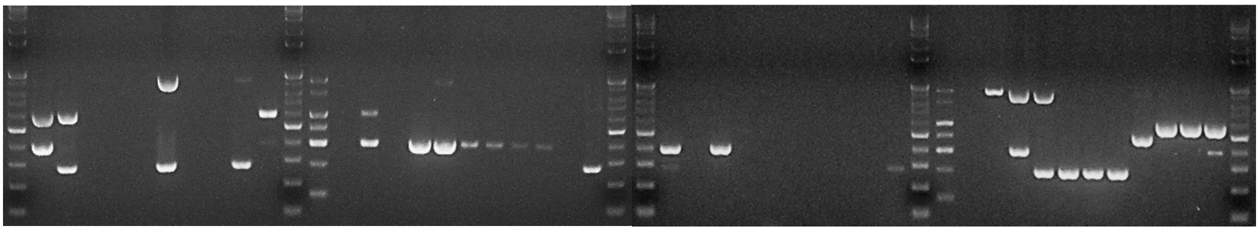

	M	1	2	3	4	5	6	7	8	9	10	M	PC	NC	11	12	13	14	15	16	17	18	19	20	M	M	21	22	23	24	25	26	27	28	29	30	M	PC	NC	31	32	33	34	35	36	37	38	39	40	M
943bp PPV5							+			+								+																							+	+	+								
800bp PPV7																																																			
590bp PPV4		+	+								+				+																																	+	+	+	
497bp PPV3																																															+				
394bp PPV6		+	+								+				+		+	+	+	+	+	+					+		+												+									+	
282bp PPV2			+				+			+														+			+									+						+	+	+	+						
162bp PPV1																																																			

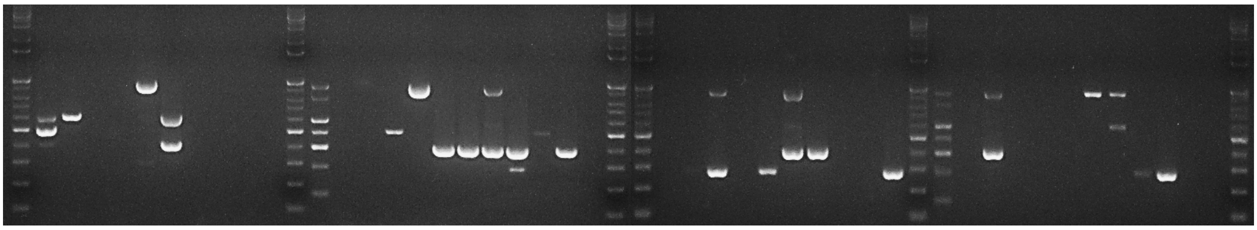

	M	41	42	43	44	45	46	47	48	49	50	M	PC	NC	51	52	53	54	55	56	57	58	59	60	M	M	61	62	63	64	65	66	67	68	69	60	M	PC	NC	71	72	73	74	75	76	77	78	79	80	M
943bp PPV5						+	+										+			+									+			+								+				+	+						
800bp PPV7																																																			
590bp PPV4		+	+				+													+												+													+						
497bp PPV3		+														+						+																													
394bp PPV6		+					+											+	+	+	+		+									+	+							+											
282bp PPV2						+															+								+		+					+										+	+				
162bp PPV1																																																			

B

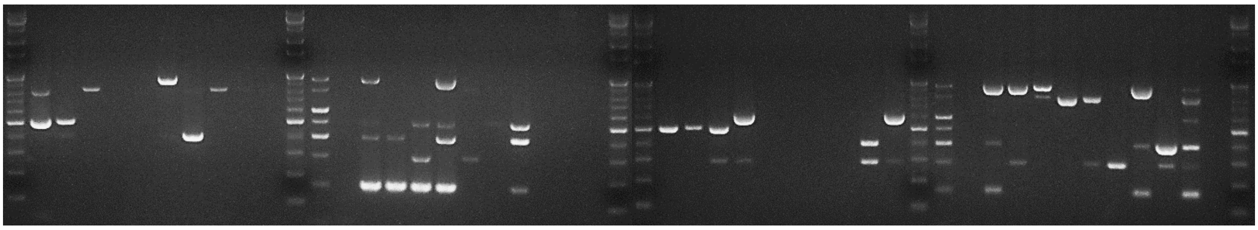

	M	1	2	3	4	5	6	7	8	9	10	M	PC	NC	11	12	13	14	15	16	17	18	19	20	M	M	21	22	23	24	25	26	27	28	29	30	M	PC	NC	31	32	33	34	35	36	37	38	39	40	M
943bp PPV5							+								+			+																						+	+	+				+		+		
800bp PPV7		+		+					+										+																							+	+	+				+		
590bp PPV4																														+						+												+		
497bp PPV3		+	+												+		+	+		+	+						+	+	+																					
394bp PPV6			+				+	+							+	+		+			+														+					+						+		+		
282bp PPV2																	+		+										+	+					+	+					+			+	+			+		
162bp PPV1															+	+	+	+			+																			+						+		+		

C

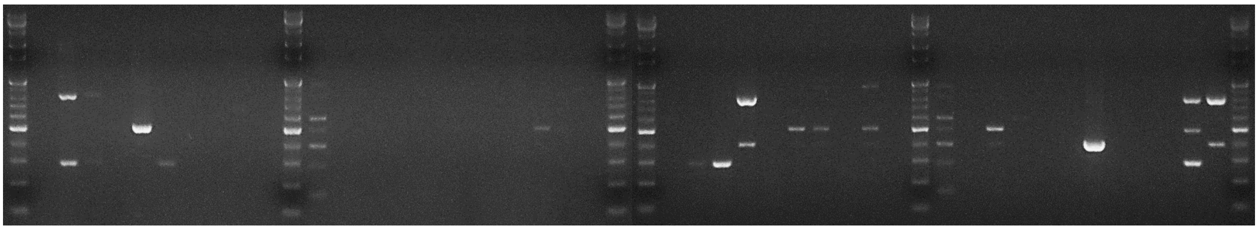

	M	1	2	3	4	5	6	7	8	9	10	M	PC	NC	11	12	13	14	15	16	17	18	19	20	M	M	21	22	23	24	25	26	27	28	29	30	M	PC	NC	31	32	33	34	35	36	37	38	39	40	M
943bp PPV5																																			+															
800bp PPV7			+	+																										+																		+	+	
590bp PPV4																																									+									
497bp PPV3						+																+										+	+		+					+								+		
394bp PPV6																														+										+				+					+	
282bp PPV2			+	+			+																					+	+																			+		
162bp PPV1																																																		
